# Comparative Study of Natamycin Encapsulation in Liposomes: Thin-Film vs. Proliposome Methods for Enhanced Stability, Controlled Release, and Efficacy Against Milk Spoilage and Pathogenic Microorganisms

**DOI:** 10.3390/foods14173064

**Published:** 2025-08-30

**Authors:** Natalija Čutović, Petar Batinić, Tatjana Marković, Jovana Petrović, Milena Obradović, Branko Bugarski, Aleksandra A. Jovanović

**Affiliations:** 1 Institute for Medicinal Plants Research “Dr. Josif Pančić”, Tadeuša Košćuška 1, 11000 Belgrade, Serbia; pbatinic@mocbilja.rs (P.B.); tmarkovic@mocbilja.rs (T.M.); 2 Department of Plant Physiology, Institute for Biological Research “Siniša Stanković”—National Institute of Republic of Serbia, University of Belgrade, Bulevar Despota Stefana 142, 11000 Belgrade, Serbia; jovana0303@ibiss.bg.ac.rs; 3 Institute for Technology of Nuclear and Other Mineral Raw Materials, Bulevar Franše d’ Epere 86, 11000 Belgrade, Serbia; m.obradovic@itnms.ac.rs; 4 Faculty of Technology and Metallurgy, University of Belgrade, Karnegijeva 4, 11000 Belgrade, Serbia; 5 Institute for the Application of Nuclear Energy INEP, Banatska 31b, 11080 Belgrade, Serbia; ajovanovic@inep.co.rs

**Keywords:** antifungal agent, food industry, encapsulation, stability

## Abstract

The aim of this study was to evaluate liposomal particles as a potential delivery system for natamycin, a widely known antimicrobial agent used in the food industry. The goal was to prolong its diffusion into the surrounding medium. Natamycin-loaded liposomes were prepared using two methods (proliposome and thin-film) and two different phospholipid mixtures. The characterization of natamycin-loaded liposomes was performed in terms of their chemical composition (FT-IR analysis), encapsulation efficiency (EE), and antimicrobial potential against spoilage and pathogenic microorganisms that can be found in milk and milk products. During the 60-day storage period, their size, polydispersity index (PDI), and zeta potential were measured. The in vitro release kinetics of natamycin from liposomes were also assessed, and the results showed a significantly lower release rate of the drug when it was encapsulated. EE showed a high level of natamycin encapsulation (>80%), which was confirmed with FT-IR analysis. The stability study indicated that these systems were stable over a 60-day storage period, as the zeta potential of all formulations was ~−25 mV. Satisfactory antimicrobial performance of the developed liposomes against *Listeria monocytogenes*, *Yersinia enterocolitica*, *Candida tropicalis*, *Candida parapsilosis*, and *Aspergillus flavus* (MIC values from 0.00625 to 4 mg/mL) indicates that loading of natamycin into liposomal carriers was an adequate method for their encapsulation and delivery in the milk industry.

## 1. Introduction

Natamycin, also known as pimaricin, natafucin, and pimafucin, is a polyene macrolide antibiotic with an empirical formula of C_33_H_47_NO_13_. Due to the fact that it is produced in submerged cultures of certain *Streptomyces* strains, nowadays, its most widely known name is natamycin. According to the nomenclature guidelines established by the World Health Organization (WHO), antibiotics derived from *Streptomyces* species are required to bear the suffix ‘mycin’ in their names [1,2]. Natamycin is known for its widespread antimicrobial activities, as it is effective against a wide variety of fungi, yeasts, and some algae species, but it is also a good antibiotic, with proven low toxicity towards the cells of mammals, both in vitro [3] and in vivo [2]. Natamycin is proven to be effective against fungal strains such as *Aspergillus* spp. [4], *Fusarium* spp. [5], *Candida* spp. [6], *Penicillium* spp. [7], and some dematiaceous fungi [6], as well as bacterial strains, such as *Escherichia coli* [8], *Staphylococcus aureus* [8], *Streptomyces lydicus* [9], etc. The use of natamycin is also widespread in the food industry, as an antimycotic agent in cheese and meat production, but also in the process of preparing wines and fruit juices. When used in the milk and meat industries, it has two main functions, i.e., prolonging the storage stability of the products, thus lowering the economic losses, and preventing the development of mycotoxins [10,11]. Mycotoxins are low-molecular-weight, toxic chemical substances produced by molds. Unlike bacterial toxins, which are protein-based and can be recognized by the immune system through antigens, mycotoxins do not elicit an antibody-mediated immune response, thus leading to potential threats to human health, like food poisoning [12]. In the case of wine and juice production, it is used as a replacement for sorbic acid and some other antifungal agents, as it is very effective in low doses (~400 times more effective than potassium sorbate) [13].

The main limitation for the use of pure antimicrobial agents, in this case, natamycin, in the food industry is the rapid diffusion of the active components from the surface, right into the mass of the product [14], lessening the efficiency of the agent over a prolonged period, therefore leading to the need for the development of novel systems for the delivery of the antimicrobial agents. For this reason, many research groups are opting for encapsulation of active agents, whether they are extracts, essential oils, drugs, antioxidants, enzymes, etc., into particles, as it has been proven to be a suitable way of overcoming the problem of high release rates [15,16,17]. Active compounds can be encapsulated using various methods, which can be split into physical, chemical, and physicochemical methods, depending on the available equipment and intended use.

Liposomes, which are amongst the most widely used encapsulation formulations, are formed when lipid molecules, such as phospholipids, organize into enclosed vesicles in an aqueous environment [18]. They can be prepared using various techniques, including the thin-film hydration method (Bangham method), reverse-phase evaporation, proliposome method, ether or ethanol injection method, and detergent removal method [19]. Emulsification combined with dilution, i.e., the proliposome method, is often used for the encapsulation of antimicrobial agents, as it is a cost-effective and rapid method suitable for large-scale production, allowing the production of liposomal vesicles of various sizes depending on specific application needs [20]. The main advantage of the proliposome method is that it allows for a higher input of agitation energy, thus leading to the formation of vesicles that are smaller in size and more homogeneous [20], which eliminates the requirement for a separate size reduction stage in the processing protocol [21]. On the other hand, the main limitation is that, despite offering significantly improved encapsulation efficiency, it lacks reproducibility when used to produce small batches of liposomes, pointing to the fact that it can easily be used for large-scale liposome production [22]. The thin-film method is a straightforward approach that is compatible with a wide range of lipid mixtures, as well as the active components intended for encapsulation, enabling its broad application. However, it has some drawbacks, as it leads to the formation of large, multilamellar vesicles, which need size reduction; organic solvents are used in the first step, which needs to be carefully removed in their entirety, prolonging the evaporation process, and making it more costly [23,24].

The literature data imply that the encapsulation of natamycin into different carriers has been the objective of various research groups [25,26,27,28,29,30,31]. The researchers focused on encapsulating natamycin into nanoemulsions, solid lipid nanoparticles (SLNs), nanostructured lipid carriers (NLCs), cyclodextrin inclusion complexes, and cell-penetrating peptides, and all of them led to a sustained release of natamycin (from 8 to 24 h) and improved corneal/vaginal penetration. On the other hand, all of these formulations have numerous limitations, such as the high cost of preparation, short shelf life (low stability), and the possibility of leading to eye or skin irritations [32,33]. Liposomal particles, as an uninvestigated option for the encapsulation of natamycin, offer the possibility to overcome the disadvantages encountered in the case of previous attempts to encapsulate natamycin. Phospholipid mixtures, used for their preparation, are very compatible with human cells, whether they be skin and ocular cells, or the cells of the organs in the gastrointestinal tract, and do not lead to irritation, while also allowing an enhanced uptake of the encapsulated drug and prolonging its release and shelf life by protecting it from the influence of its surroundings.

Liposomal encapsulation has been widely explored for improving stability, solubility, and controlled release of bioactive compounds in food and pharmaceutical systems. While both thin-film hydration and proliposome methods have been applied for various antimicrobials, there is currently no direct, standardized comparison of these techniques for natamycin encapsulation under identical formulation conditions. As mentioned above, natamycin, as a potent antifungal agent approved for use in food preservation, possesses relatively low water solubility and potential for rapid diffusion into surrounding media, which limit its efficacy in prolonged storage applications. In this study, we address this gap by systematically evaluating the impact of preparation method and phospholipid composition on the physicochemical characteristics, efficiency of the encapsulation process, release kinetics, and antimicrobial performance of natamycin-loaded liposomes. A distinctive feature of our approach is the incorporation of in vitro release testing using a Franz diffusion cell, which enables simulation of diffusion-controlled release through a membrane interface. This method offers more realistic insights into natamycin’s potential behavior in food matrices compared with conventional bulk-release assays. By integrating 60-day stability monitoring with application-relevant conditions for milk and dairy products, this work provides the first comprehensive analysis of thin-film versus proliposome methods for natamycin delivery, offering practical formulation insights for the food industry.

## 2. Materials and Methods

### 2.1. Standards and Reagents

Natamycin Tredemix^®^ (Natamycin 50%) was purchased from Biokom Trendafilov Company, Belgrade, Serbia. Phospholipon 90H (a commercial lipid mixture, containing hydrogenated phospholipids, with a content of ≥90.0%; its basic structure consists of a glycerol backbone, fatty acid chains (which are saturated due to hydrogenation, thus providing higher stability), a phosphate group, and a choline head group [34]); Lipoid S100 (a commercial lipid mixture, with a soybean phosphatidylcholine content of ≥94.0%; its structure consists of a polar head group (choline) linked to a glycerol backbone with two fatty acid chains (one of which is saturated, and the other unsaturated [35])), and cholesterol (≥99%) were purchased from Lipoid^®^ Company, GmbH, Ludwigschafen, Germany. Ethanol (95%, *v*/*v*), methanol, and chloroform were obtained from Zorka Pharma, Šabac, Serbia. Tryptic Soy Broth (TBS) was from Torlak Institute (Belgrade, Serbia). *p*-iodonitrotetrazolium violet, sodium phosphate monobasic (≥99%), and sodium phosphate dibasic dihydrate (≥98%) were purchased from Sigma-Aldrich, Schnelldorf, Germany.

### 2.2. Liposomal Particles Preparation Using Thin-Film Method

Liposomes containing natamycin were prepared using the thin-film method [36,37], with some modifications. The liposomes were prepared using two phospholipid mixtures: Lipoid S 100 and Phospholipon 90H. In short, when Lipoid S100 served as the base lipid, a mixture containing 0.125 g of cholesterol, 1 g of phospholipids, and 30 mg of natamycin (drug/phospholipid = 1:33.3) was dissolved in 5 mL of a chloroform/methanol solution (2:1, *v*/*v*). The resulting solution was then transferred to a 50 mL round-bottom flask. The solvents were evaporated under a vacuum of 100 mm Hg using a rotary vacuum evaporator (IKA^®^-WERKE, HB4 basic, IKA, Staufen, Germany) at 45 °C to form a thin film at 600 rpm. Evaporation continued for approximately 3 h until a dry residue was formed. The organic solvent is eliminated slowly by this method to yield a thin lipid film on the interior surface of the flask. The film was then hydrated with 30 mL of phosphate-buffered solution (PBS) with a pH of 7.4, for 60 min at 45 °C, at a stirring rate of 800 rpm. In the case when Phospholipon 90H was used as the phospholipid mixture, the same procedure was followed, with the difference in the set temperature, as Phospholipon 90H requires a temperature of ~65 °C to transform into a liquid state. The thin film of Phospholipon 90H was hydrated using 30 mL of PBS for 60 min at 45 °C and a stirring rate of 800 rpm. As a control, plain liposomes (without natamycin) were also made using the same protocol.

### 2.3. Liposomal Particles Preparation Using the Proliposome Method

The liposomes containing natamycin were also prepared using the proliposome encapsulation method [15], with some modifications. The liposomes were prepared using the same two phospholipid mixtures as for the thin-film method. Briefly, to prepare the lipid mixture, 0.9 g of Lipoid S100 phospholipids, 0.150 g of cholesterol, and 30 mg of natamycin (drug/phospholipid = 1:30) were dissolved in 1.5 mL of ethanol of 96% *v*/*v*, and maintained at 45 °C. After the mixture thickened, due to the evaporation of ethanol, the aqueous phase was incorporated (30 mL) using small amounts, and the mixture was stirred at 800 rpm for a duration of 60 min at 25 °C. On the other hand, when Phospholipon 90H was used as the phospholipid mixture, the same procedure was followed, with the difference in the set temperature, as it was ~65 °C in this case (during ethanol evaporation and homogenization of lipids and natamycin), while the mixture was hydrated and stirred at 800 rpm for 60 min at 25 °C. As a control, plain liposomes (without natamycin) were also prepared using the same protocol.

### 2.4. Determination of the Encapsulation Efficiency

The natamycin-loaded liposome particles were separated from the non-encapsulated fraction via centrifugation at 17,500 rpm and 4 °C for 45 min (Centrifuge 5430R, Eppendorf^®^, Hamburg, Germany). The EE was determined by measuring the concentration of natamycin in the supernatant using BioTek Epoch 2 Elisa Microplate Spectrophotometer (Agilent Technologies, Santa Clara, CA, USA) at a wavelength of 304 nm. EE was calculated according to the concentration of natamycin present in the supernatant, obtained after centrifugation, as shown in Equation (1):

(1) $$EE\;\left[\%\right]=\frac{C_{i}-C_{sup}}{C_{i}}\cdot 100\%$$

*C_i_* denotes the initial concentration of the natamycin solution utilized for liposome formulation, whereas *C_sup_* denotes the natamycin concentration measured in the supernatant after centrifugation. Natamycin (0.02–1 mg/mL) was used as a standard for making the calibration curve (equation of the calibration curve: y = 18.3x + 1.071, with the linear regression at R^2^ = 0.9931). The results of EE were expressed as a percentage. The measurement was performed in triplicate, and the data are presented as mean ± standard deviation.

### 2.5. Fourier Transform Infrared Spectroscopy

The chemical interactions between the phospholipid mixtures, cholesterol, and natamycin for all of the prepared liposomes were characterized via FT-IR spectroscopic analysis. The FT-IR spectra were collected using a Thermo Scientific Nicolet iS50 spectrophotometer (Thermo Fisher Scientific, Waltham, MA, USA). FT-IR spectra were recorded using Attenuated Total Reflection (ATR) with a diamond ATR smart accessory, in the range of 4000–400 cm^−1^ at 64 scans per spectrum and 2 cm^−1^ resolution. For the FT-IR measurements, the liposomes were dried in an inert atmosphere at ~30 °C for 10 h.

### 2.6. Particle Size, Size Distribution, Zeta Potential, and Storage Stability

The particle size, polydispersity index (PDI), and zeta potential of the prepared liposomes were measured for 60 days, following preparation using the Malvern Zetasizer Nano ZS (Malvern Instruments, Worcestershire, UK). During the 60-day stability evaluation, the liposomal system was stored in the refrigerator at 4 °C. The measurement was repeated three times, and the data are presented as mean±standard deviation.

### 2.7. Controlled Release Study

The study of the controlled release of natamycin from natamycin-loaded liposomes, as well as a natamycin solution, was performed using the Franz diffusion cell (PermGear, Inc., Hellertown, PA, USA). The donor and acceptor compartments of a Franz cell are divided by a cellulose acetate membrane (Permgear, Hellertown, PA, USA) with a diffusion area of 4.91 cm^2^ and a pore size of 0.2 µm. The donor compartment (0.05 g, d = 2.5 cm) contained the samples. A magnetic stirrer was used to continuously mix the release media (PBS, pH = 7.4, c = 0.1 mol/L) at 37 °C and 400 rpm in the receptor compartment [38]. For the duration of 24 h, samples were collected at set times. All samples taken from the receptor compartment were replaced with 0.5 mL of fresh receptor medium, which was thermostated at 37 °C, to preserve synchronized operational parameters. Natamycin concentration was calculated for these samples. The results of each analysis were statistically processed after being run three times.

The release studies were further analyzed to determine the diffusion coefficients and overall mass transfer resistance of natamycin released from the liposomes. The diffusion coefficients were calculated according to the method described by Pjanović et al. [39], based on Fick’s second law, as shown in Equation (2):

(2) $$\ln\left[\frac{C_{D}^{0}-C_{R}^{0}}{C_{D}-C_{R}}\right]=D\cdot \beta \cdot t$$

where *C_D_* and *C_R_* represent the concentrations of natamycin in the donor and receptor compartments, respectively, at time *t*, while and $C_{R}^{0}$ are the corresponding concentrations at *t* = 0. *D* is the diffusion coefficient, and *β* (2.49 × 10^4^ m^−2^) is a geometrical constant for a standard Franc diffusion cell (20 mL) [39]. The total mass transfer resistance (*R*) was calculated using the following Equation (3):

(3) $$R=\frac{\delta }{D}$$

In this equation, *δ* refers to the effective membrane thickness, which includes both the acetate–cellulose membrane and the layer (height) of liposomes, while *D* is the diffusion coefficient obtained from Equation (2). The value of *R* thus represents the combined resistance of the acetate–cellulose membrane and the liposomes.

The control for the release study was prepared by dissolving 50 mg of natamycin in 50 mL of PBS to prepare a solution for obtaining the diffusion profile, with the same concentration of natamycin in the system as in the liposomes.

For liposomes, the Higuchi kinetic equation, derived from Fick’s second law of diffusion, which describes the mass transfer of active compounds, i.e., natamycin from a controlled-release system, is commonly applied (Equation (4)).

(4) $$\frac{m_{t}}{m_{\infty }}=k\cdot t^{1/2}$$

where $m_{t}$ and $m_{\infty }$ represent the mass of natamycin in the receptor compartment of the Franz diffusion cell at time *t* and at infinite time (∞) respectively, while *k* is the kinetic release constant, which includes the coefficient of diffusion [39]. OriginPro 9 and MS Excel 2020 software were used for fitting the results (R^2^ ≥ 0.95) and the calculation of kinetic coefficient values [40,41].

### 2.8. Measurement of the Antimicrobial Potential of Natamycin-Loaded Liposomes

#### 2.8.1. Measurement of the Antibacterial Potential of Natamycin-Loaded Liposomes

Antibacterial activity was tested against three bacterial strains from the American Type Culture Collection (ATCC). For the bioassays, two Gram-positive bacterial species, *Listeria monocytogenes* (ATCC 19111) and *Bacillus cereus* (clinical isolate), and one Gram-negative bacterial species, *Yersinia enterocolitica* (ATCC 9610), were used. All bacterial strains used in the test were from the Laboratory of Mycology, Department of Plant Physiology, University of Belgrade, Institute for Biological Research “Siniša Stanković”, National Institute of the Republic of Serbia. To assess the antibacterial activity of the natamycin-loaded liposomes, a modified version of the microdilution method was used [42]. Bacterial species were cultured for 24 h at 37 °C in a TSB medium. The fresh bacterial suspension was adjusted with TSB to a final concentration of 1.0 × 10^8^ per well. All natamycin-loaded liposomes had a natamycin concentration of 1 mg/mL. Minimal inhibitory concentration (MIC) and minimal bactericidal concentration (MBC) assessments were performed by a serial dilution method in 96-well microtiter polystyrene plates. The MIC is defined as the lowest concentration of an antimicrobial agent that inhibits visible growth of a microorganism under standardized in vitro conditions. The MIC is identified as the lowest concentration at which no visible microbial growth is observed after the incubation period. MIC and MBC values were determined by serial dilution of natamycin-loaded liposomes into microtiter plates containing broth (the wells contained 160 μL of liposomal suspension and 40 μL of medium). Then, bacterial suspension (10 μL) was added to each well, followed by a 24 h incubation at 37 °C. After incubation, *p*-iodonitrotetrazolium violet (0.04 mL, 0.2 mg/mL) was introduced, and the plates were incubated at 37 °C for an additional 30 min. The MIC was defined as the lowest sample concentration that reduced in color intensity (light red compared to the deep red in untreated controls) or complete absence of color [23]. MBC was determined after serial sub-cultivation, with 2 μL from each well being transferred to fresh broth and incubated for 24 h at 37 °C. The MBC was the lowest concentration that eradicated bacterial growth, indicating a 99.5% reduction in the initial inoculum. A natamycin solution with a concentration of 1 mg/mL was used as a positive control, while blank liposomes were used as negative controls. The measurement was performed in triplicate, and the data are presented as mean ± standard deviation.

#### 2.8.2. Measurement of the Antifungal Activity of Natamycin-Loaded Liposomes

Antifungal activity was tested against two *Candida* strains and one *Aspergillus* strain from the American Type Culture Collection (ATCC). For the bioassays, *Candida tropicalis* (ATCC 750), *Candida parapsilosis* (ATCC 22019), and *Aspergillus flavus* (ATCC 9643), which all cause milk and milk product spoilage, were used. All fungal strains used in this assay were from the Laboratory of Mycology, Department of Plant Physiology, University of Belgrade, Institute for Biological Research “Siniša Stanković”, Belgrade, Serbia. The antifungal assay was conducted by the modified EUCAST procedure [43], which was previously described by Stojković et al. [44]. A natamycin solution with a concentration of 1 mg/mL was used as a positive control, while blank liposomes were used as negative controls. The measurement was performed in triplicate, and the data are presented as mean±standard deviation.

### 2.9. Statistical Analysis

The statistical analysis was performed using one-way analysis of variance (one-way ANOVA) and Duncan’s *post hoc* test within the software STATISTICA 7.0. The differences were deemed statistically significant at *p* < 0.05, n = 3.

## 3. Results

In the present study, liposomes with an antimicrobial agent, natamycin, were developed using thin-film and proliposome methods, followed by their characterization in terms of EE, particle size, size distribution, zeta potential, storage stability, and antimicrobial potential. Natamycin release kinetics from the solution and developed liposomes were tested using the Franz diffusion cell, while their chemical composition was analyzed using FT-IR spectroscopy.

### 3.1. Encapsulation Efficiency

**Figure 1:**
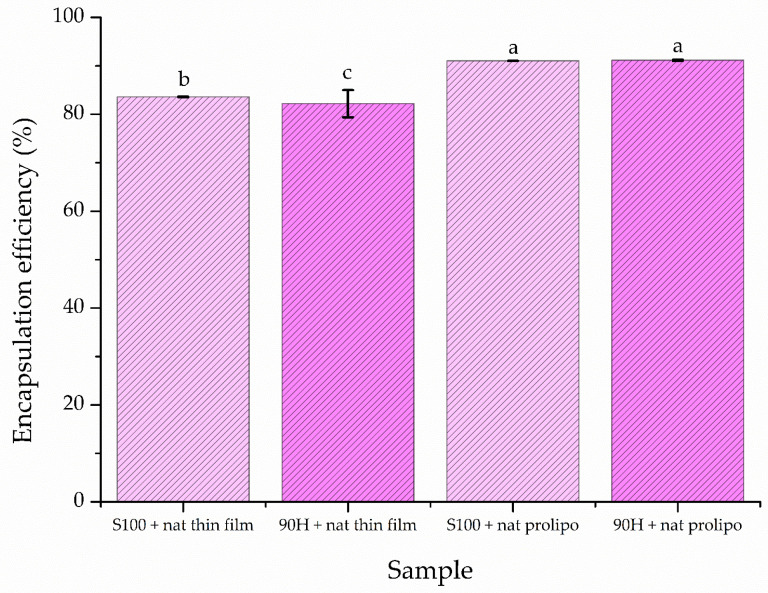
Encapsulation efficiency of developed liposomal formulations with natamycin using thin-film and proliposome techniques and Lipoid S100 and Phospholipon 90H phospholipids; different letters show a statistically significant difference (*p* < 0.05; n = 3, one-way ANOVA, analysis of variance, Duncan’s *post hoc* test); data are presented as mean ± standard deviation, n = 3.

EE is one of the most important parameters that characterizes the encapsulation process [45]. The EE of natamycin into liposomal particles, using two phospholipid mixtures and two methods, is presented in Figure 1. The level of entrapment of natamycin was assessed right after the preparation of liposomes.

The EE of natamycin into liposomes prepared using the proliposome method was 91.02 ± 0.01% (for Lipoid S100-based liposomes) and 91.14 ± 0.13% (for Phospholipon 90H-based ones). On the other hand, the encapsulation efficiency for the liposomes developed by the thin-film method was significantly lower, i.e., 83.57 ± 0.06% (for Lipoid S100) and 82.19 ± 2.80% (for Phospholipon 90H). Due to the shown successful encapsulation of natamycin into liposomes, further physicochemical characterization of the developed liposomes was performed.

### 3.2. FT-IR Spectra

**Figure 2:**
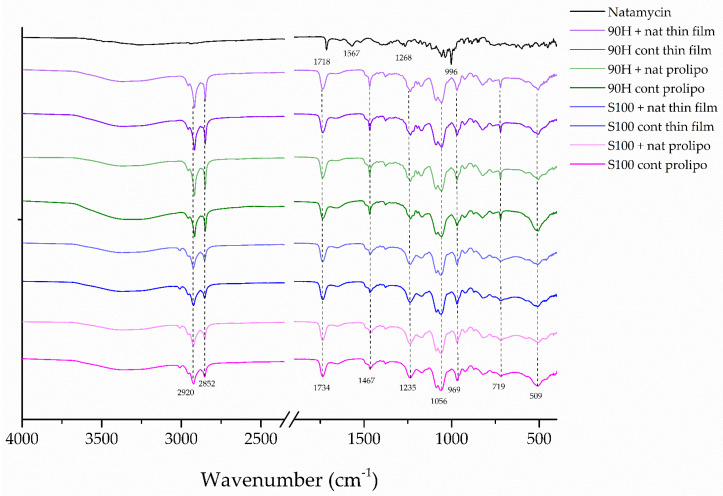
FT−IR spectra of the natamycin, plain (blank), and natamycin-loaded liposomes prepared using thin-film and proliposome techniques, and Lipoid S100 and Phospholipon 90H phospholipids.

The observed high EE levels show successful encapsulation of natamycin into liposomal vesicles composed of Lipoid S100 and Phospholipon 90H phospholipids, suggesting potential for their future application. However, it is essential to analyze potential interaction, incompatibility, or creation of new functional groups between natamycin and employed phospholipids. Therefore, FT-IR spectroscopy was applied with the aim to deeper understand eventual changes that can occur between active compound and carrier during encapsulation. Namely, the presence of characteristic interactions between natamycin and the phospholipids/cholesterol mixtures was analyzed by FT-IR spectroscopy. Figure 2 shows the FT-IR spectra of natamycin-loaded and blank (control) liposomes.

While long-term stability determines liposomes’ practical applicability, after the analysis of the obtained FT-IR spectra, the physical characterization of liposomes with natamycin and their storage stability were examined.

### 3.3. Physical Properties of Developed Liposomes and Their Storage Stability

**Figure 3:**
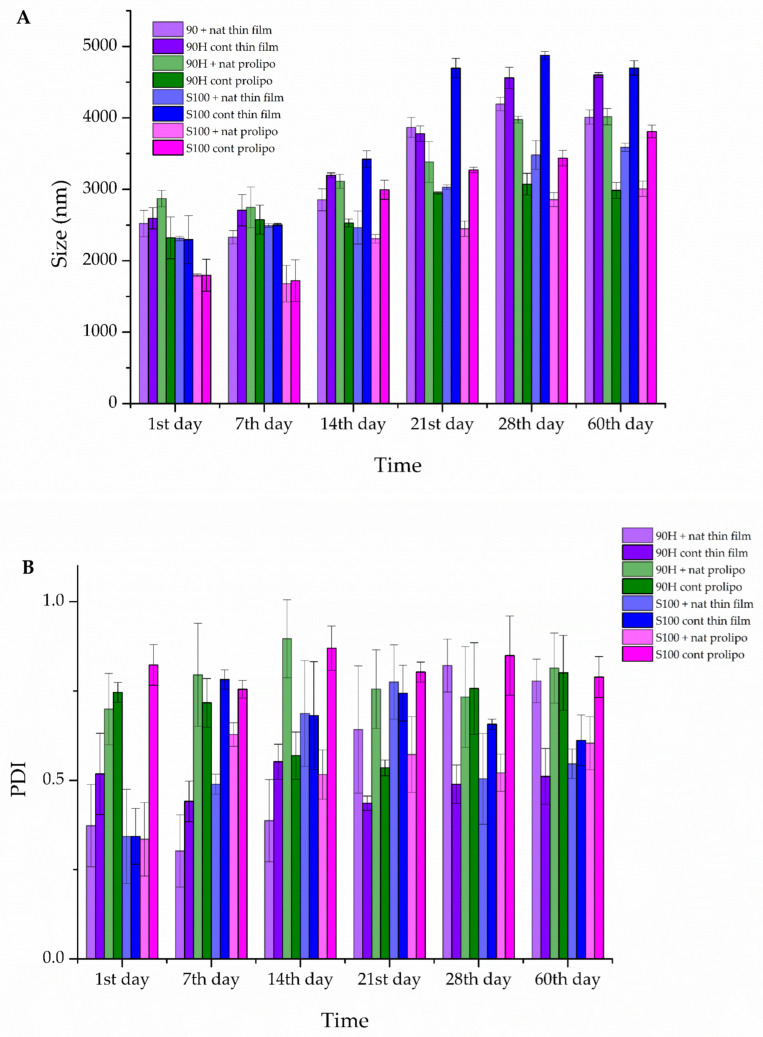

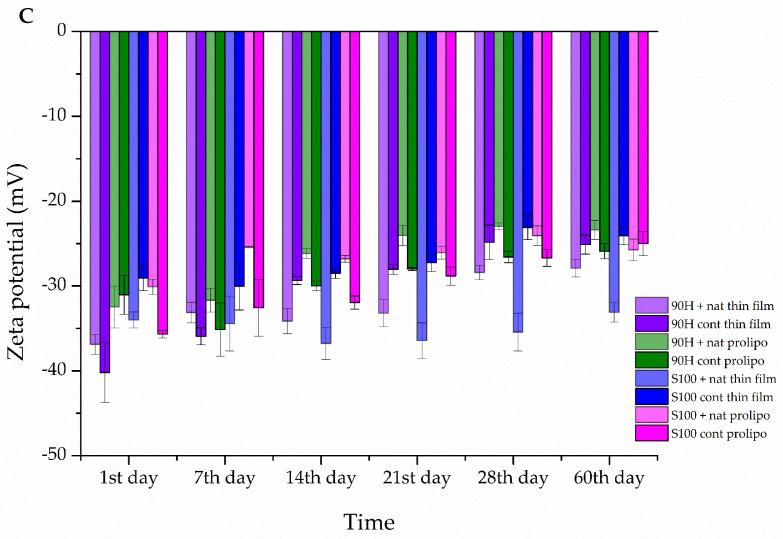
Liposome size (**A**), polydispersity index (PDI) (**B**), and zeta potential (**C**) of the blank (control) and natamycin-loaded liposomes prepared using thin-film and proliposome techniques and Lipoid S100 and Phospholipon 90H phospholipids, during 60 days of storage at 4 °C; the data are presented as mean ± standard deviations, n = 3.

Since the physical properties of developed liposomes (size, PDI, and zeta potential) and liposomes’ stability during storage are crucial for their functional performance, the stability of liposomal vesicles with natamycin was evaluated using dynamic light scattering. Particle size, PDI, and the zeta potential were evaluated for 60 days to test the storage stability of the blank (control) and natamycin-loaded liposomes, and the results are presented in Figure 3A–C. The measurements were performed on the 1st, 7th, 14th, 21st, 28th, and 60th day.

As can be seen from the graph in Figure 3A, plain and natamycin-loaded liposomes prepared using Lipoid 100S and the proliposome method possessed the lowest diameter (1797.0 nm and 1795.3 nm, respectively) compared to other developed liposomes, whose diameters varied in a range of ~2300 nm to ~2900 nm. In all Lipoid 100S liposomal samples, the proliposome technique provided the particles with a lower size in comparison to the particles obtained in the thin-film method (2296.0 nm for plain liposomes and 2313.0 nm for natamycin-loaded liposomes). Additionally, the incorporation of natamycin did not cause changes in lipid vesicle size, except in the case of Phospholipon 90H liposomes prepared using the proliposome protocol, where the presence of natamycin increased particle diameter.

In the case of the monitoring of vesicle size over time, the smallest changes during the 60 days were detected in the case of the control (blank) liposomes prepared using the proliposome method and Phospholipon 90H as the lipid base, as the size changed from 2321.0 nm to 2985.7 nm (Figure 3A). On the other hand, in the case of liposomal formulations containing natamycin, the smallest liposome size after 60 days was in the case of the liposomes prepared using Lipoid S100 as the phospholipid mixture and the proliposome technique (3007.3 nm). However, the increase in diameter of the mentioned particles was very high, ~1000 nm, as in the case of natamycin-loaded Phospholipon 90H liposomes obtained in the proliposome method. A noticeable growth in the particle size values for all of the prepared formulations (natamycin-loaded and blank (control) ones) can be seen at the 60-day mark (Figure 3A), as they almost doubled in size in comparison to the 1st day. PDI values of all developed liposomes are presented graphically in Figure 3B. The data obtained varied between different samples and encapsulated compounds in the mentioned parameter, proving the significant influence of the employed preparation method and phospholipids. Specifically, the highest PDI values, *i.e*., the highest heterogeneity of the system, were measured in the plain Lipoid 100S and Phospholipon 90H samples prepared using the proliposome technique (0.823 and 0.746, respectively). The lowest values were obtained for the plain Lipoid 100S liposomes developed in the thin-film method and liposomes with natamycin (0.335–0.373), except in the case of natamycin-loaded Phospholipon 90H liposomes prepared using the proliposome technique (0.699).

The values of size distribution varied during the 60-day storage period, particularly in the case of plain Lipoid 100S liposomes obtained in the thin-film technique and natamycin-loaded liposomes (except Phospholipon 90H liposomes prepared using the proliposome technique) (Figure 3B). In the case of the control (blank) liposomes prepared using both preparation methods and Phospholipon 90H, as well as Lipoid 100S and the proliposome technique, there was no significant increase in the PDI values during storage. The most significant change between the PDI value of the liposomes on the day of their preparation and after a 60-day storage period was in the case of the natamycin-loaded liposomes prepared using the thin-film method and Phospholipon 90H, as the increase was ~120%, thus presenting the mentioned sample as susceptible to sedimentation. However, as can be seen in Figure 3B, all of the prepared formulations stayed somewhat homogeneous even after 60 days, as the PDI value only slightly changed in comparison to the 28-day mark, and none of them reached a value of 1.

As the high absolute values of zeta potential (positive or negative) of the prepared liposomal formulations imply that the systems are electrostatically stabilized, which leads to a prolonged period without aggregation, therefore making them longer lasting, the zeta potential values were measured during 60 days of storage. Figure 3C depicts the zeta potential on the day of liposome preparation, as well as its changes over the 60-day period. The highest negative ζ potential value was detected in the case of the control (blank) liposomes prepared using the thin-film method and Phospholipon 90H (−40.20 ± 3.54 mV), making them the most electrostatically stable upon production, but having the biggest decrease in the absolute value after 60 days (−25.11 ± 1.12 mV), making them the least stable during prolonged storage. Only in the case of natamycin-loaded liposomes prepared using the thin-film method and Lipoid S100 the absolute value of zeta potential did not change after the 60-day mark (−34.00 ± 0.95 mV on the 1st day and −33.09 ± 1.15 mV on the 60th day) pointing to the possibility that the natamycin leads to improved electrostatic stability, as absolute value of the zeta potential of the control parallel decreased during this period (from −29.07 ± 1.46 mV to −24.05 ± 1.07 mV).

In this study, natamycin-loaded liposomes were successfully prepared using Lipoid S100 and the proliposome technique, achieving high encapsulation efficiency and favorable stability over 60 days at refrigerated conditions. In addition, our results indicate that both preparation methods are suitable for laboratory-scale encapsulation.

Due to proven high EE, the absence of incompatibility between natamycin and used phospholipids (shown via FT-IR spectra), and the determined physical characteristics and stability of liposomal vesicles, which can suggest potential for controlled release, the mentioned parameter was examined using a Franz diffusion cell and a model for the calculation of diffusion coefficients and resistance.

### 3.4. Controlled Release Study Data

**Figure 4:**
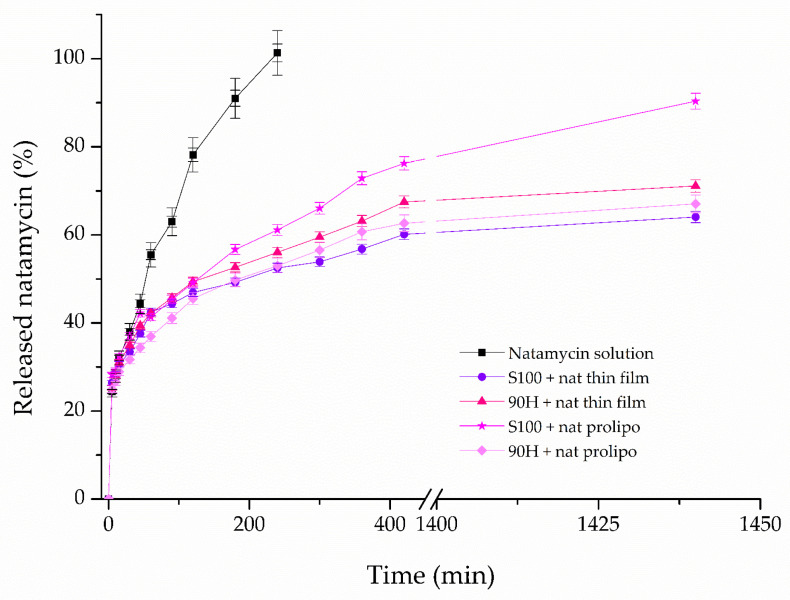
Release profiles of natamycin from natamycin solution, and liposomes prepared using thin-film and proliposome techniques, and Lipoid S100 and Phospholipon 90H phospholipids, expressed as the percentage of released natamycin; the data are presented as mean ± standard deviations, n = 3.

Given the physicochemical properties of the developed liposomes and data on their stability, the next evaluation was the release behavior of natamycin from the liposomes. The Franz diffusion cell was used in order to measure the resistance that the liposomal particles represent for the mass transfer of pure natamycin in the release study. Figure 4 shows the values of released natamycin in percentages, and they are presented as a function of time, for the duration of a 24 h period. All prepared liposomal formulations containing natamycin were subjected to the assessment of their prolonged release, while the natamycin solution was used as a control.

The natamycin release experiments were conducted for 24 h using a static Franz diffusion cell. The release profiles of natamycin from liposomes were compared with the release profile of free natamycin, which served as a control.

The percentage of released natamycin from five different formulations in the receptor compartment depicted in Figure 3 was calculated using Equation (5):

(5) $$Released\;natamycin,\;\%=\frac{m_{NAT\left(t\right)}}{m_{NAT\left(eq\right)}}\times 100\%$$

where $m_{NAT\left(t\right)}$ stands for natamycin released at time *t*, and $m_{NAT\left(eq\right)}$ is the amount of natamycin released at equilibrium.

As evident from the results, lower natamycin release was observed in liposomal formulations prepared with Phospholipon 90H using both liposome preparation methods, with approximately 65% released from thin-film liposomes and about 60% from those prepared by the proliposome method after 1440 min, indicating that carriers based on Phospholipon 90H provide more controlled (and prolonged) release of the drug compared to their Lipoid S100-based counterparts.

**Figure 5:**
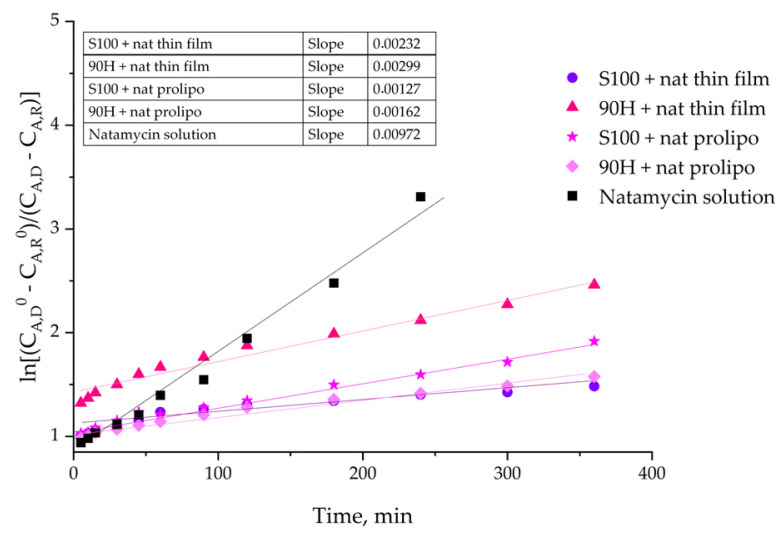
Dimensionless plot of natamycin concentration as a function of time for the release curves.

The diffusion coefficients were calculated using the slope of the linear portion of the curve shown in Figure 5, while the diffusion resistance represents the ratio between the sample thickness and the diffusion coefficient (Table 1).

In Table 1, it can be observed that the Lipoid S100 + natamycin formulation obtained by the thin-film method had the least mass transfer of the active component (natamycin) from the donor to the receptor compartment of the Franz diffusion cell, and consequently, the highest diffusion resistance.

The coefficients of diffusion and the resistance to the diffusion that is produced by the liposomal bilayer were calculated based on the data collected during the release study (Figure 4), and the results are presented in Table 1. The total diffusion resistance across the membrane can be calculated from the known values of membrane thicknesses, *δ* (Table 1, column 2), and diffusion coefficients, *D* (Table 1, column 3), using Equation (3) as described in Section 2.5. The calculated total diffusion resistance (R) presented in Table 1, column 4, represents the cumulative resistance of a semipermeable cellulose acetate membrane and the resistance of the liposome membrane [46,47]. The contribution of the resistance, which is generated by the synthetic membrane, is determined from the diffusion of free natamycin (Table 1, row 5, column 4). The resistance from only liposomes (R_LIP_) can be calculated from a subtraction of the membrane resistance from the total diffusion resistance (Table 1, column 5).

The kinetic analysis of natamycin release from different liposomal formulations obtained in this study was also performed. Specifically, the Higuchi kinetic model was applied, and the results are presented in Figure S1 and Table S1. The release profiles of natamycin from the tested formulations showed high consistency with the Higuchi model, indicating that the release process was predominantly controlled by molecular diffusion. The linear relationship between the cumulative release and the square root of time further supports Fickian diffusion as the primary mechanism controlling natamycin transfer from liposomes into the receptor compartment of the Franz diffusion cell.

Also, the main criterion used to evaluate the predictive accuracy applied model was the R^2^ value. In general, as the value of R^2^ is closer to 1, the applied kinetic model precisely predicts values [40]. According to our findings, most of the analyzed liposomal formulations met these criteria. Therefore, both thin-film- and proliposome-based delivery systems exhibited a prolonged release profile compared to the natamycin solution, confirming their potential for industrial application.

While controlled and prolonged release of bioactives and long-term stability are crucial for functional performance, thus, the antimicrobial potential of formulated liposomes with natamycin was evaluated using major milk spoilage microorganisms.

### 3.5. Antimicrobial Potential of Natamycin-Loaded Liposomes

Since the above-mentioned findings suggest that the liposomes are suitable carriers for natamycin, prompting further investigation into their antimicrobial capacity, this experiment aimed to prove natamycin’s antibacterial and antifungal activity after encapsulation within liposomal vesicles.

#### 3.5.1. Antibacterial Potential of Natamycin-Loaded Liposomes

**Figure 6:**
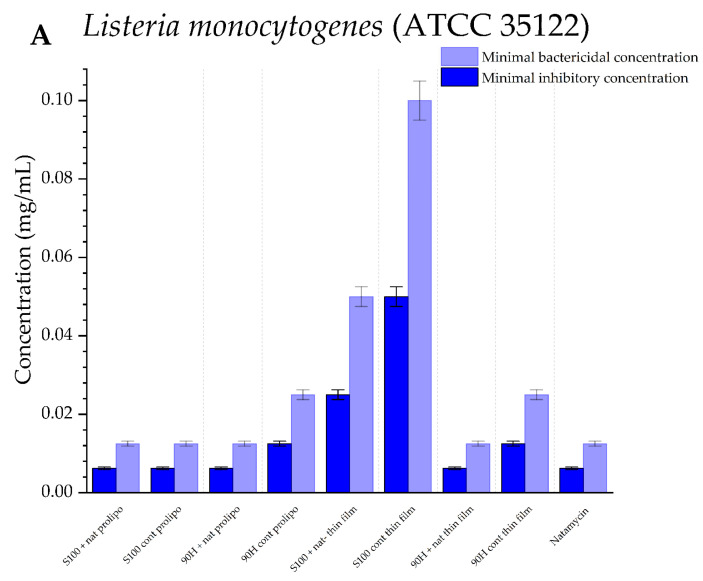

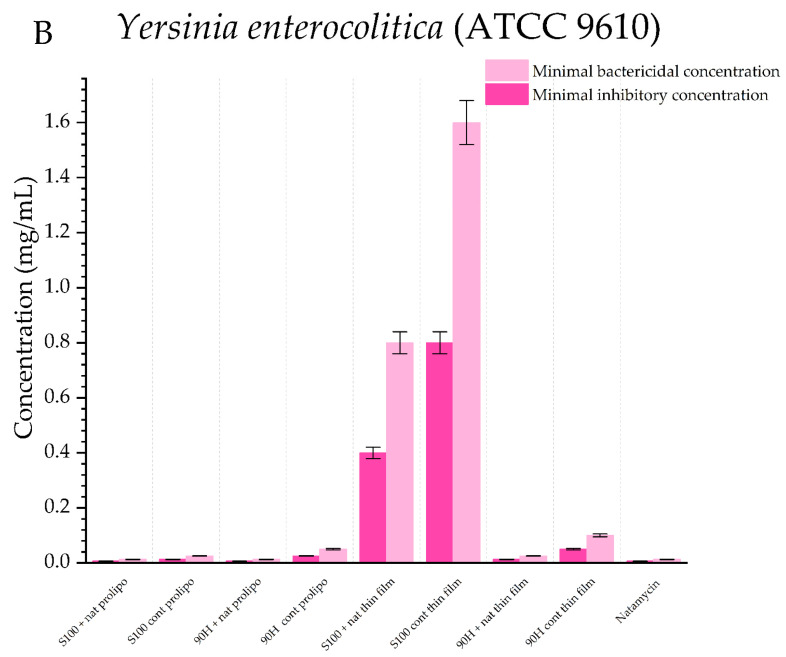
Antibacterial activity of natamycin, blank (control), and natamycin-loaded liposomes prepared using thin-film and proliposome techniques, and Lipoid S100 and Phospholipon 90H phospholipids, against pathogenic bacteria: (**A**) *Listeria monocytogenes* and (**B**) *Yersinia enterocolitica*, expressed as minimal inhibitory and minimal bactericidal concentrations (MIC and MBC, respectively); the data are presented as mean±standard deviations, n = 3.

Natamycin is widely known as an antimicrobial agent used in the food industry, as it is effective against foodborne, food spoilage, and pathogenic bacteria. Figure 6 presents the results of the antibacterial potential of blank (control) and natamycin-loaded liposomes against *L. monocytogenes* and *Y. enterocolitica*, which are pathogenic bacteria. The prepared liposomal formulations were also assessed in terms of their activity against *B. cereus*, a food spoilage pathogen; however, they showed no potential in inhibiting the growth of this pathogen in any of the tested concentrations.

To assess the antibacterial effects of the phospholipid mixtures and cholesterol, the blank liposomes were subjected to this assay, and the Lipoid S100 control liposomes obtained by the proliposome method were the most effective against both bacterial strains. In terms of the natamycin-loaded liposomes, *Y. enterocolitica* was more susceptible to their effect, and a complete eradication of its growth was achieved with lower concentrations of the liposomes containing natamycin (MBC values ranged from 0.0125 to 0.1 mg/mL), whereas *L. monocytogenes* was more resilient to their influence (MBC was from 0.0125 to 1.6 mg/mL). The lowest concentrations needed for the inhibition of the growth of *Y. enterocolitica* were for the liposomal formulations obtained by the proliposome method, with both Lipoid S100 and Phospholipon 90H (MIC values were 0.00625 mg/mL). In the case of *L. monocytogenes*, there was no such difference in the effectiveness of the liposomes prepared using the different methods to encapsulate natamycin. The MIC values were equal (0.00625 mg/mL) for both formulations prepared by the thin-film method, as well as the formulation obtained by the proliposome method, using Phospholipon 90H, with the only exception being the natamycin-loaded liposomes prepared using Lipoid S100 and the proliposome method, as the MIC value was 4 times greater in that case.

#### 3.5.2. Antifungal Potential of Natamycin-Loaded Liposomes

**Figure 7:**
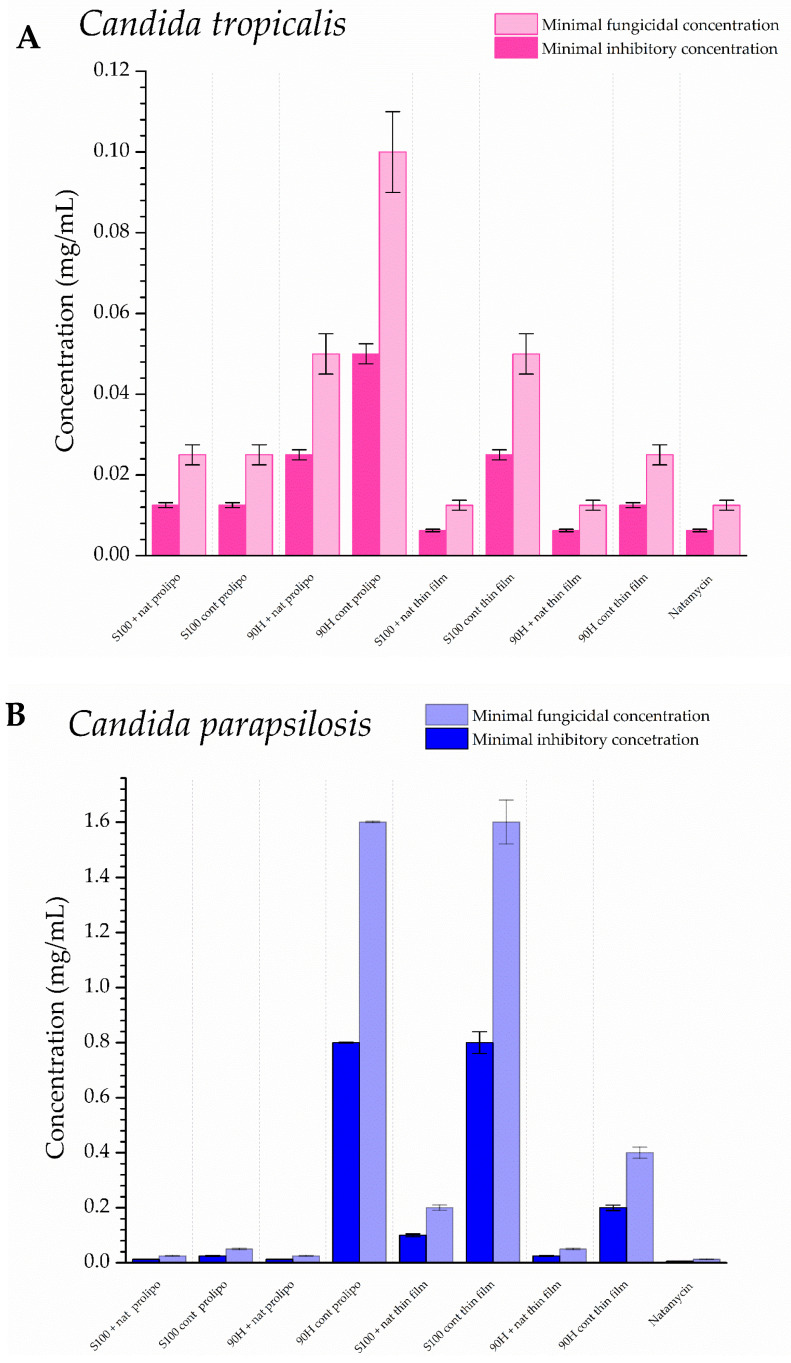

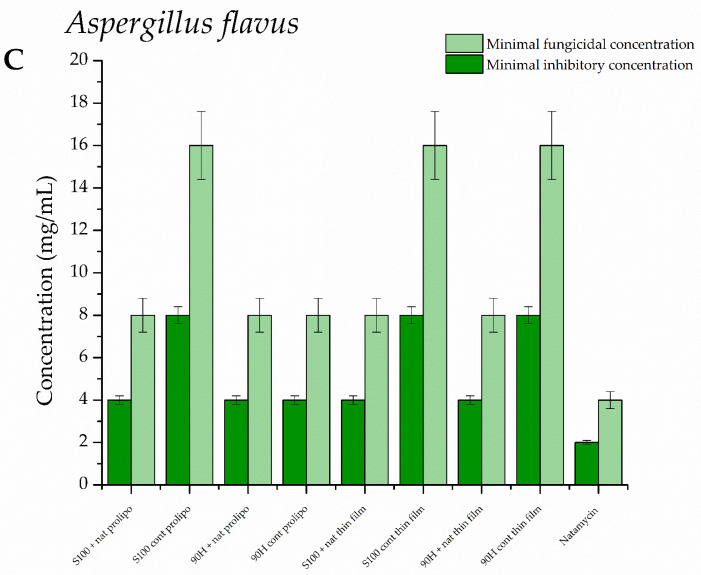
Antifungal activity of natamycin, blank (control), and natamycin-loaded liposomes prepared using thin-film and proliposome techniques, and Lipoid S100 and Phospholipon 90H phospholipids against milk and milk product spoilage fungi: (**A**) *Candida tropicalis*, (**B**) *Candida parapsilosis*, and (**C**) *Aspergillus flavus*, expressed as minimal inhibitory and minimal fungicidal concentrations (MIC and MFC, respectively); the data are presented as mean ± standard deviations, n = 3.

All prepared liposomes were tested against *A. flavus*, as well as *Candida* strains (*C. tropicalis* and *C. parapsilosis*), which are all fungal pathogens associated with spoilage in dairy products (Figure 7).

A natamycin solution of the identical concentration of natamycin as in the liposomes was used to assess the susceptibility of the tested strains to the pure antifungal agent.

The difference in the antifungal potential of natamycin in its free state, in comparison to when it is encapsulated into liposomes, is clearly depicted in Figure 7, with the information about the activity of blank (control) liposomes as well. Both tested *Candida* species were equally susceptible to the influence of natamycin solution, with an MFC of 0.0125 mg/mL, which was the lowest of the tested concentrations. For *C. tropicalis*, the MFC values of natamycin-loaded liposomes ranged from 0.0125 to 0.05 mg/mL, while for the blank (control) ones, they were between 0.025 and 0.1 mg/mL. On the other hand, in the case of *C. parapsilosis*, the values were somewhat higher, as the MFC ranged from 0.025 to 0.2 mg/mL for natamycin-loaded liposomes and from 0.05 to 1.6 mg/mL for blank (control) ones. On the other hand, in the case of *A. flavus*, the MIC values were significantly higher, as they were 4 mg/mL for all of the natamycin-loaded liposomes, whereas for the control (blank) parallels, they ranged from 4 to 8 mg/mL.

The highest MFC value against *C. parapsilosis* was determined for the liposomes prepared using the thin-film method and Lipoid S100 as the lipid mixture to encapsulate natamycin, which could be brought into connection with the slowest release of the active component, which was shown in Section 3.3. On the other hand, in the case of *C. tropicalis*, the lowest inhibition of fungal growth was detected in the case of natamycin-loaded liposomes, developed by the proliposome method, using Phospholipon 90H as the lipid base. This could be due to the higher transition temperatures of this lipid mixture, which caused a low amount of the active agent to be released at 37 °C, as it needs a temperature of ~65 °C.

## 4. Discussion

### 4.1. Encapsulation Efficiency

According to the information present in the literature, it is possible to encapsulate different antifungal agents into liposomes, with an encapsulation efficiency of 23–88.1%. The efficiency depends on the drug used and the encapsulation method employed [48,49,50,51]. In the present study, an easy and affordable proliposome method and a more widely used, but also more costly, thin-film method were employed to encapsulate natamycin into the lipid matrices. Both methods can efficiently encapsulate a range of substances, including antimicrobial agents, vitamins, plant extracts, and essential oils, whether hydrophilic or hydrophobic. This is due to the unique structure of the liposomal particles, which can entrap target compounds either in the surrounding water or between the phospholipid tails, depending on their solubility [52]. A significant difference in encapsulation efficiency of natamycin appeared only when the liposomes were developed using different methods (thin-film and proliposome techniques. When the same preparation method was used with different phospholipid mixtures, only minimal variation was observed. The difference that occurs when the proliposome method is used, in comparison to the thin-film method, could be due to a longer period of hydration, as natamycin is poorly soluble in water; thus, prolonged exposure to heating during the hydration phase might have elevated the amount of natamycin that was able to dissolve, thus being more available for encapsulation [11]. The difference between the encapsulation efficiency of the same phospholipid mixture, depending on the method used, may be due to differences in the chemical composition of the used phospholipid mixtures (Section 2.1). Lipoid S100 has both unsaturated and saturated fatty acid chains with shorter chains, thus leading to the production of smaller liposomes (as shown in Figure 3A), with a higher EE, in the case of the proliposome method (denser packing). On the other hand, the Phospholipon 90H contains only unsaturated fatty acids (Section 2.1), with longer chains, thus leading to the formation of bigger liposomal vesicles (Figure 3A), due to the packing order of natamycin into the fatty acid chains, resulting in a higher EE in the case of the proliposome method.

### 4.2. FT-IR Spectra

All FT-IR spectra presented in Figure 2 contain the pair of peaks at 2852 and 2920 cm^−1^ of C—H bonds in =CH_2_ and —CH_3_ groups in the unsaturated alkyl chains of the phospholipid molecule [38]. It is notable that the encapsulation of natamycin into liposomes led to a decrease in peak intensity in the sample obtained by encapsulating natamycin into Phospholipon 90H-based liposomes using the proliposome method compared to the control(blank) parallel. This indicates reduced mobility and increased ordering of the alkyl chains, suggesting hydrophobic interactions between the polyene segment of natamycin and the fatty acid tails of the phospholipid molecules. The peak at 1734 cm^−1^ in the spectra of all liposomal formulations can be attributed to the carbonyl stretching vibration (C=O) of the ester bonds presented in the fatty acid chains of phospholipids [15,17]. Namely, natamycin is assumed to be located within the phospholipid bilayer of liposomes, with its hydrophobic polyene chain embedded among the fatty acid tails and its hydrophilic parts facing the aqueous interface [53]. The peak observed at 1718 cm^−1^ can be attributed to the carbonyl (C=O) stretching vibration of the ester group in the macrolide ring of natamycin. The absorption peak at 1567 cm^−1^ is associated with CH=CH stretching vibrations in the polyene chain and asymmetric stretching of the deprotonated carboxylate (COO^−^) groups in natamycin [2]. The band at 1467 cm^−1^ can be attributed to the scissoring vibrations of the =CH_2_ groups in the fatty acid chains [16]. The absorption peak at 1268 cm^−1^ can be ascribed to the asymmetric stretching vibration of the C–O–C bond in the epoxide group of the natamycin [2]. The band centered at 1235 cm^−1^ can be attributed to the asymmetric and symmetric stretching vibrations of the PO_2_^−^ groups [16,38]. A P–O–C stretching band, which represents the phosphodiester bond in the phospholipid headgroup, was detected at 1056 cm^−1^. A sharp peak at approximately 996 cm^−1^ (broad range 1010–990 cm^−1^) can be attributed to out-of-plane bending vibrations of CH=CH groups in the conjugated polyene chain of the natamycin [54]. The vibrational modes related to the quaternary ammonium group of the choline moiety [(CH_3_)_3_N^+^–CH_2_–CH_2_–OH] are responsible for the band at 969 cm^−1^. These bands most likely involve asymmetric and symmetric C–N stretching and related vibrations [54]. According to the study by Jovanović et al. [16], the band observed at 719 cm^−1^ can be assigned to the symmetric bending vibration of the C–N bond of the choline moiety. In the study by Cacela and Hincha [55], vibrations of the phosphate group in phospholipids were observed in a broad region around 600–400 cm^−1^, including bending modes of the PO_4_ group, with a peak detected at 509 cm^−1^. FT-IR analysis confirmed not only the presence of natamycin in the lipid bilayer, but also suggested hydrophobic interactions between natamycin and the lipid carrier.

### 4.3. Physical Properties of Developed Liposomes and Stability Study

Previous studies have demonstrated that the diameter of the liposomal vesicles is strongly influenced by both the composition of the liposomes and the nature of the encapsulated material [56,57]. However, in the case of liposomes loaded with different antimicrobial agents, such as nisin [58], pediocin AcH [59], and calcein [60], no significant difference in particle size was observed between loaded and unloaded liposomes. According to the findings in the literature, the thin-film method, which is based on the formation of a lipid thin film due to the solvent evaporation from the mixture containing the drug and the lipid mixture, followed by the hydration process, and thus the liposomes are formed, leads to the formation of multilamellar vesicles, that significantly vary in sizes, and usually require size reduction [61,62]. The size of the blank (control) liposomes and the natamycin-loaded ones in this study follows the findings. The highest vesicle size detected in the case of the thin-film method was expected, as this method is usually used for the preparation of larger liposomes. On the other hand, the proliposome method, which is characterized by a high energy input of agitation, results in the production of smaller, unilamellar vesicles [20]. The disagreement that occurs between these findings and the results of this study can be linked to the use of different phospholipid mixtures for the liposome preparation, as the difference in the composition is linked to the presence of lipid chains of various lengths, thus leading to the formation of different-sized vesicles. It is important to state that the size of the liposomal vesicles increased slowly up until the 28-day mark, and then exponentially grew up to the 60-day mark. This rapid growth could be brought into connection with the presence of cholesterol in all of the prepared formulations, which facilitates fusion and swelling, as is directly related to the membrane fluidity [63].

The PDI value is a direct measurement of the distribution of sizes in a system [64]. The PDI values that were obtained in this study align with the data reported in the literature, with the PDI values decreasing with the encapsulation of natamycin into liposomes, as did happen in the case of encapsulating Paclitaxel [65], letrozole [66], curcumin [67], resveratrol [68], etc., into liposomes. Lipid concentration, composition, liposome preparation method, and mixing duration all influence the dispersity of the liposomal suspension [69]. Trucillo et al. [70] observed that higher bioactive compound loading resulted in a wider range of dispersions and, thus, increased PDI values. Besides this, it has previously been reported that the use of a single phospholipid, instead of a phospholipid mixture, can result in better uniformity in the vesicle size distribution [71].

The zeta potential is a critical parameter for assessing the repulsive forces between liposomal particles and, consequently, the stability of the liposomal system, as it leads to electrostatic stability. According to available literature, a zeta potential value of approximately −30 mV or +30 mV in a liposomal suspension is indicative of its adequate stability over extended storage periods, as it prevents particle aggregation and sedimentation [15,44,72].

In the present study, the zeta potential varied in all the prepared liposomal suspensions during the 60-day stability study. After the 21st day, the zeta potential started to decrease continuously, reaching stable values by day 60. They attained approximately the same values, pointing to their electrostatic stability during storage. The changes in the surface charge values observed in this study suggest that aggregation is unlikely over a 60-day storage period. This duration is typical for many liquid milk products and some cheeses, where natamycin is used, as it has been shown to extend shelf life by up to 4 weeks [11,73,74]. This leads to the conclusion that the encapsulation of natamycin into liposomes may aid in an even longer storage stability.

In this study, colloidal stability was assessed through dynamic light scattering-derived measurements of particle size, PDI, and zeta potential, all of which are widely accepted indicators in liposomal and lipid nanoparticle research [75]. These parameters are widely recognized as reliable indicators of liposomal stability, with changes in size and PDI over time serving as sensitive markers of aggregation or fusion events. Zeta potential provides insight into electrostatic repulsion between particles; the more negative or positive the value, the greater the colloidal stability, with ±30 mV often cited as a threshold for moderate to good stability [44].

Direct visualization techniques such as transmission electron microscopy (TEM) or cryo-TEM, which could confirm morphological stability and detect subtle aggregation, were not performed, presenting a limitation of the present study. Incorporating these methods in future work would strengthen the mechanistic understanding of long-term liposomal stability and aggregation of liposomal particles.

### 4.4. Controlled Release Study

The controlled release and diffusion profiles of natamycin from natamycin-loaded liposomes were compared with the diffusion profile of natamycin aqueous solution that was used as a control. Also, the release profiles demonstrate a controlled (and prolonged) release of natamycin from liposomes, following a typical controlled drug release pattern. Diffusion of natamycin from the control solution occurred rapidly. The concentration of natamycin in the receptor compartment reached a maximum approximately after 180 min, which is expected due to the immediate availability of the free drug in solution without any diffusion resistance or barrier limitations.

Hence, a slower release rate of natamycin from the liposomes was to be expected. In contrast, natamycin release from liposomes and its consequent diffusion through the acetate-cellulose membrane proceeded at a notably slower rate. Consequently, after ~400 min, a steady state was reached for all of the prepared liposome formulations. The diffusion of natamycin into the acceptor compartment of the Franz diffusion cell continued at a slower rate, indicating equilibrium between donor and acceptor compartments. These results suggest that liposomes can serve as effective carriers for the controlled and sustained release of natamycin, which is a critical consideration for practical applications in the dairy industry. Concerning the impact of the specific lipids that were used in the preparation of liposomes, as well as the employed method, there are apparent differences in the kinetics of natamycin release from them. The liposomes prepared by the proliposome method, using Lipoid S100 as the lipid base, had a more fluid membrane, thus allowing for easier release of natamycin, which could be due to the interactions between the used lipid mixture and cholesterol [76]. The slower release of natamycin from the other liposomal formulation prepared by the proliposome method could be connected to the higher transition temperatures of the fatty acids present in it. This led to more stable liposomal particles, with a more rigid membrane. As a result, only ~67% of the loaded natamycin was released after 24 h [77].

On the other hand, when the liposomes used for the encapsulation of natamycin were prepared using the thin-film method, the situation was reversed, i.e., the higher release rate was detected in the case of Phospholipon 90H lipid mixture. The release rate was similar for these two formulations up until the 30 min mark, and it was ~45%, after which it was somewhat faster from the Phospholipon 90H-based formulation. After a period of 24 h, a release of ~64% of natamycin was detected for liposomes where Lipoid S100 was used, which could be because it is made up from soybean lecithin, which is a solid lipid mixture, leading to the formation of a stiffer membrane, thus halting the release of the active compound [15].

During liposome formation, physicochemical interactions, such as hydrophobic interactions, hydrogen bonding, and van der Waals forces between natamycin and lipid components, are more pronounced in Phospholipon 90H-based liposomes. Their tighter packing contributes to stronger retention of natamycin compared to the more fluid Lipoid S100-based systems. The reduced release rate observed in Phospholipon 90H formulation may be attributed to the higher degree of saturation and elevated phase transition temperature of hydrogenated phospholipids. These factors enhance bilayer order and decrease permeability. This has been observed in hydrogenated soy phosphatidylcholine liposomes, which demonstrated tighter membrane packing and superior storage stability [78], while more saturated, long-chain phospholipids form bilayers that act as highly effective barriers against small molecules [79]. These observations are supported by established biophysical principles, showing that saturation increases chain rigidity and transition temperature, whereas unsaturation introduces flexibility and increases permeability. These results demonstrate the potential of liposomes as effective systems for the controlled and prolonged release of natamycin, which is a key aspect for practical applications.

The release kinetics of natamycin varied significantly across formulations. The solution of free natamycin showed the highest slope value (9.72 × 10^−3^) and consequently the fastest drug release (D = 7.02 × 10^−9^ m^2^/s). Namely, the natamycin from liposomes is released more slowly. The thin-film liposomes based on Phospholipon 90H exhibited approximately 3-fold higher release compared to those based on Lipoid S100. On the other hand, liposomes prepared using the proliposome method with the Lipoid S100 phospholipid mixture reduced the release rate by approximately 45% compared to the corresponding thin-film liposomes. All liposome samples provided higher overall natamycin diffusion resistance compared to the solution, promoting prolonged release. To the best of the authors’ knowledge, there are currently no studies on liposome-encapsulated natamycin that report comparable results in terms of release kinetics and diffusion resistance calculations, as presented in our study.

Given the differences in the permeability shown by the liposomal bilayer, it can be concluded that different liposome preparation methods and different lipid mixtures used to encapsulate natamycin would differ in the means of the diffusion resistance. The highest diffusion resistance is calculated in the case of the liposomes prepared by the thin-film method, using Lipoid S100 as the lipid mixture, which agrees with the results of the slowest release rate of natamycin from these liposomes. On the other hand, the lowest resistance to the natamycin diffusion was shown by the liposomes with the fastest rate of releasing the drug, *i.e.*, the liposomes prepared by the proliposome method, using Lipoid S100 as the base. This points to the importance of choosing the right method for the preparation of liposomes for encapsulating this specific drug, depending on whether the goal is faster or slower release in the medium.

Our results indicate that both preparation methods (thin-film and proliposome techniques) are suitable for laboratory-scale encapsulation and controlled release of natamycin. Namely, these methods, using food-grade components, are based on mild processing conditions (avoiding high shear/temperature) and compatible with existing dairy product manufacturing steps, which may facilitate scale-up. These findings highlight the potential of food-grade liposomal carriers for dairy preservation. However, considerations such as industrial-scale reproducibility, cost-effectiveness, and impacts on sensory attributes (e.g., taste, texture, and appearance) remain to be evaluated. Future studies addressing these aspects will be essential to translate laboratory observations into practical, commercial applications in the dairy industry.

### 4.5. Antimicrobial Activity

#### 4.5.1. Antibacterial Activity

All the natamycin-loaded liposome formulations were effective in inhibiting the growth of the two bacterial strains that lead to milk and milk product spoilage, chosen for this study. The results indicated that incorporation of natamycin into the liposomes led to a decrease in its antibacterial activity only when the thin-film method was used for their development. This could be due to a lower amount of natamycin encapsulated in the liposomes using the thin-film method (Figure 1), resulting in less antimicrobial agent available to affect the bacteria. To be more precise, *Y. enterocolitica* was more resilient to the effects of natamycin-loaded liposomes prepared using this method. According to the results, the MIC values of the liposome formulations containing natamycin were lower for Gram-positive bacteria (*L. monocytogenes*). This is because Gram-positive bacteria have a thick peptidoglycan layer in their cell walls, which is a target for natamycin. Natamycin’s primary path of functioning is by binding to the cell walls of Gram-positive bacteria, which are more accessible to the compound [80].

The natamycin solution (used as the positive control) and all of the liposomal formulations were ineffective in suppressing the growth of the Gram-positive bacteria *B. cereus*. This is consistent with the existing literature findings, as natamycin is known to be an antifungal agent with limited antibacterial activity [11,81].

However, in Gram-negative bacteria, the inner membrane is surrounded by a double-layered membrane made of lipopolysaccharides, which often acts as a barrier to permeability [82]. The hydrophobic nature of natamycin plays into the basis for the molecular pathway of the substance, for its antibacterial activity, as the drug is directly connected to the bacterial cell wall [83].

The lipid components of the liposomes interact with the hydrophobic metabolites produced by the bacteria, inhibiting their growth. This slows the growth of bacteria even by the blank (control) liposomes [84]. In the case when natamycin is encapsulated into liposomes, the micelles are formed around the hydrophobic natamycin, keeping it out of direct contact with the bacterial cell walls. This leads to a prolonged antibacterial effect, as the amount of the released drug grows in the system. Furthermore, the higher antibacterial potential of the liposomes prepared by the proliposome method could be explained by a higher EE (EE ~91% for proliposome formulations, compared to ~83% for thin-film formulations), and also by the differences in the zeta potential. A slightly lower zeta potential of the liposomes prepared using the proliposome method leads to enhanced electrostatic attraction to the negatively charged bacterial cell walls, thus leading to this disruption [85]. While the results of this study demonstrate sustained antibacterial effects of natamycin-loaded liposomes, direct evidence linking these effects to slow release from the liposomes is not presented. However, such sustained activity may be influenced by the physicochemical properties of liposomes, including vesicle size, surface charge, lipid composition, and encapsulation efficiency, which are known to modulate release profiles and interactions with bacterial membranes [86,87]. Future studies including long-term release experiments are needed to confirm this relationship.

#### 4.5.2. Antifungal Activity

Lipid mixtures, or phospholipids, are found to be suitable carriers for various antimicrobial agents. Their lipid bilayer can mimic cell membranes, allowing for the active compound to be released on the inner part of the target microorganism [15].

Natamycin has long traditional uses as an antifungal agent, but has only recently gained attention for food preservation. This is especially relevant as concerns about microbial spoilage and public health threats in the food industry grow. Its extensive use in various food matrices, including dairy, highlights a growing demand for effective preservatives that are in line with consumer standards [88]. Given the diversity of food matrices and spoilage microorganisms, numerous delivery systems and combinations have been developed to enhance the application of natamycin.

This was explored in a study by Meena et al. [11], where 8 ppm natamycin most effectively reduced yeast growth without significantly affecting key quality parameters (pH, acidity, sugars), making it the optimal dose for preserving vanilla-flavored yogurt over 28 days of cold storage. Results regarding anticandidal properties follow the data we presented herein.

Furthermore, efforts have been made to explore possibilities to combine natamycin with other natural products like food preservatives. Thus, rosemary extract enhanced the antifungal effect of natamycin against *A. niger* when combined [89]. Moreover, a combination of natamycin, nisin, pomegranate, and grape seed extracts into chitosan coatings effectively preserved the quality and extended the shelf life of fresh strawberries stored at 4 °C. Among the treatments, chitosan with natamycin consistently reduced microbial growth. All coatings helped prevent spoilage and off-odors, suggesting strong potential for broader food preservation applications [90]. Along with this, antimicrobial nanocomposite films enriched with natamycin and cellulose nanocrystals showed strong antifungal activity, improved mechanical strength, and successfully inhibited *Saccharomyces cerevisiae* growth in cheese, confirming their potential as active packaging for food preservation [91]. Natamycin-loaded nanoparticles have also been shown to suppress the growth of *A. flavus*, a fungal pathogen that contaminates cheese and produces harmful mycotoxins called aflatoxins [92].

In the present study, liposomes with natamycin and Lipoid S100 were successfully formulated using thin-film and proliposome procedures, providing effective antimicrobial activity against common milk spoilage organisms (*C. parapsilosis*, *C. tropicalis*, as well as *A. flavus*). Given that in our study both the proliposome- and thin-film-obtained liposomes proved effective in delivering natamycin and exhibiting antifungal activity, future research should focus on combining these delivery systems with natural bioactive compounds to further enhance stability, extend antimicrobial activity, and support the development of advanced strategies in food preservation.

## 5. Conclusions

This study optimized the encapsulation of natamycin into liposomes using the thin-film and proliposome methods, with Lipoid S100 and Phospholipon 90H as phospholipid mixtures. The proliposome method with Lipoid S100 showed the highest encapsulation efficiency and antimicrobial activity against *Candida parapsilosis*, while the thin-film method with Lipoid S100 provided the slowest release rate, ensuring prolonged natamycin release. FT-IR confirmed successful encapsulation with minimal drug leakage for all prepared formulations. The stability study indicated that liposomes prepared with Lipoid S100 remained stable for 60 days, suggesting superior stability compared to Phospholipon 90H.

Both preparation methods are suitable for laboratory-scale encapsulation and controlled release of natamycin, with the proliposome method + Lipoid S100 showing the most promise for cheese surface coating and liquid milk preservation, offering sustained antimicrobial activity. The Lipoid S100-based liposomes exhibited enhanced release kinetics and antibacterial persistence, contributing to natamycin’s application efficiency in dairy preservation.

This is the first systematic comparison of these methods and phospholipid mixtures for natamycin encapsulation, highlighting the innovation in optimization. While these findings highlight the potential of food-grade liposomal carriers for dairy preservation, considerations such as industrial-scale reproducibility, cost-effectiveness, and impacts on sensory attributes (e.g., taste, texture, and appearance) remain to be evaluated. Future studies should focus on in vivo tests and assessing the impact of food matrices on liposome stability and antimicrobial activity to advance towards practical dairy industry applications.

## Figures and Tables

**Table 1:** Coefficients of diffusion and diffusion resistance of natamycin-loaded liposomes: *δ*—thicknesses of the samples in the donor section of the Franz cell; *D*—diffusion coefficient; *R*—total mass transfer resistance; R_LIP_—resistance arising only from liposomal membrane.

Sample	*δ* (m)	*D* (m^2^/s)	*R* (s/m)	R_LIP_ (s/m)
S100 + nat, thin film	4.07 × 10^−3^	2.28 × 10^−10^	1.79 × 10^7^	1.69 × 10^10^
90H + nat, thin film		1.40 × 10^−9^	2.91 × 10^6^	2.76 × 10^10^
S100 + nat, prolipo		6.13 × 10^−10^	6.64 × 10^6^	6.31 × 10^9^
90H + nat, prolipo		2.82 × 10^−10^	1.44 × 10^7^	1.37 × 10^10^
Natamycin solution		7.02 × 10^−9^	* 5.80 × 10^5^	** n.a.

* Diffusion resistance arising from the acetate-cellulose membrane; ** not applicable.

## Data Availability

Data is contained within the article.

## Supplementary Materials

The following supporting information can be downloaded at: https://www.mdpi.com/article/10.3390/foods14173064/s1, Figure S1: Kinetic fitting of natamycin (nat) release from the tested liposomal formulations prepared by thin-film and proliposome methods and Lipoid S100 and Phospholipon 90H phospholipids, using the Higuchi model. Fitting data are represented by lines, while symbols refer to experimental data of cumulative natamycin release from liposomes; Table S1: Model parameters for natamycin release from liposomes prepared by thin-film and proliposome methods into phosphate buffer (pH 5.5) medium.

## Abbreviations

The following abbreviations are used in this manuscript:

PBS	Phosphate Buffer Solution
TSB	Tryptic Soy Broth
EE	Encapsulation Efficiency
PDI	Polydispersity Index
FT-IR	Fourier Transform Infrared
ATCC	American Type Culture Collection
MIC	Minimal Inhibitory Concentration
MBC	Minimal Bactericidal Concentration
MFC	Minimal Fungicidal Concentration
GI	Gastrointestinal
SLN	Solid Lipid Nanoparticles
NLC	Nanostructured Lipid Carriers
