# Supplementary material for: Comparative Study of Natamycin Encapsulation in Liposomes: Thin-Film vs. Proliposome Methods for Enhanced Stability, Controlled Release, and Efficacy Against Milk Spoilage and Pathogenic Microorganisms
Source: Foods. 2025 Aug 30;14(17):3064. doi: 10.3390/foods14173064 (PMC12428085; doi:10.3390/foods14173064)
Supplement: Supplementary file 1 [file foods-14-03064-s001.zip › foods-3804933-supplementary.pdf]

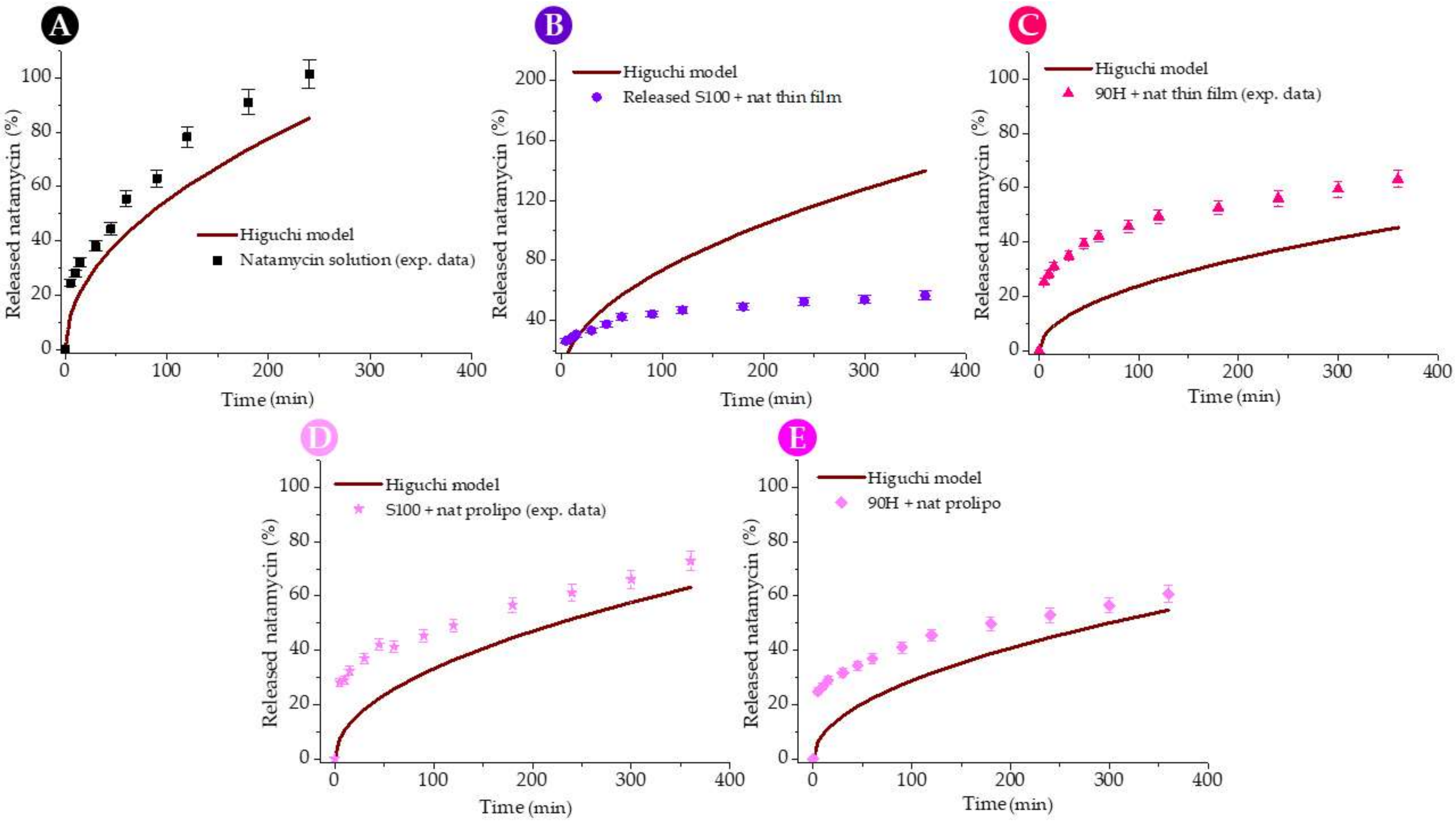

Figure S1. Kinetic fitting of natamycin (nat) release from the tested liposomal formulations prepared by thin-film and proliposome methods and Lipoid S100 and Phospholipon 90H phospholipids, using the Higuchi model. Fitting data are represented by lines, while symbols refer to experimental data of cumulative natamycin release from liposomes

Table S1. Model parameters for natamycin release from liposomes prepared by thin-film and proliposome methods into phosphate buffer (pH 5.5) medium.

Formulation	*k*	R^2
S100 + nat thin film	30.77	0.96
90H + nat thin film	9.97	0.97
S100 + nat prolipo	14.77	0.96
90H + nat prolipo	11.64	0.95
Natamycin solution	15.80	0.92
